# Waist-to-Hip Ratio and Inflammatory Parameters Are Associated with Risk of Non-Alcoholic Fatty Liver Disease in Patients with Morbid Obesity

**DOI:** 10.3390/biomedicines10102416

**Published:** 2022-09-27

**Authors:** Marta Borges-Canha, João Sérgio Neves, Maria Manuel Silva, Fernando Mendonça, Telma Moreno, Sara Ribeiro, João Correa, Catarina Vale, Juliana Gonçalves, Helena Urbano Ferreira, Sara Gil-Santos, Vanessa Guerreiro, Ana Sande, Selma B. Souto, Jorge Pedro, Paula Freitas, Davide Carvalho, CRIO Group

**Affiliations:** 1 Serviço de Endocrinologia, Diabetes e Metabolismo do Centro Hospitalar Universitário de São João, 4200-319 Porto, Portugal; 2 Departamento de Cirurgia e Fisiologia, Faculdade de Medicina, Universidade do Porto, 4200-319 Porto, Portugal; 3 Serviço de Medicina Interna do Centro Hospitalar Universitário de Cova da Beira, 6200-251 Covilhã, Portugal; 4 Serviço de Medicina Interna do Centro Hospitalar Universitário de São João, 4200-319 Porto, Portugal; 5 Serviço de Endocrinologia do Instituto Português de Oncologia do Porto, 4200-319 Porto, Portugal; 6 Investigação e Inovação em Saúde (i3s), Faculdade de Medicina, Universidade do Porto, 4200-319 Porto, Portugal

**Keywords:** NAFLD, waist circumference, waist-to-hip ratio, inflammation

## Abstract

Non-alcoholic fatty liver disease (NAFLD) is associated with several other metabolic disorders, which are typically pro-inflammatory states. Body fat content is an important marker of metabolic health and abdominal fat is associated with harmful cardiometabolic outcomes. We aimed to evaluate the association between the risk of NAFLD (through Fatty Liver Index (FLI), and BMI, AST/ALT ratio, and presence of diabetes (BARD)), and anthropometric parameters, predictors of metabolic status, in patients with morbid obesity, and to evaluate the association of FLI and BARD scores with pro-inflammatory markers. We have retrospectively studied patients with morbid obesity followed in our center. In total, 2184 participants were included, with an average age of 42.8 ± 10.6 years, 84.5% being females. We report a positive association of FLI with waist circumference (β = 0.10 [0.09 to 0.11], *p* < 0.01) and waist-to-hip ratio (β = 8.68 [6.85 to 10.52, *p* < 0.01]), even after adjusting for age, sex, body mass index, diabetes, and dyslipidemia (*p* < 0.01 for both adjusted models). The associations of BARD with anthropometric measures were significant only in the non-adjusted model. There was a positive association between both FLI and BARD and C-reactive protein. Our results point towards a positive association between waist-to-hip ratio and the risk of hepatic steatosis, and between pro-inflammatory markers and both hepatic steatosis and fibrosis.

## 1. Introduction

Non-alcoholic fatty liver disease (NAFLD) is in the spotlight of scientific research and its importance is being increasingly recognized. Its prevalence is growing in parallel to the prevalence of obesity [1]. It is a metabolic liver disease associated with several other metabolic disorders, and it is considered as the hepatic counterpart of metabolic syndrome (MS) [2,3]. NAFLD is characterized by a continuum of liver dysfunction, ranging from simple steatosis to steatohepatitis and fibrosis, cirrhosis, and even hepatocarcinoma [4,5,6,7]. Scores used to predict hepatic steatosis and fibrosis, and measurement of serum liver enzymes, are essential in clinical practice to screen and evaluate the progression of NAFLD. There are many scores available, each with its strengths and limitations. The Fatty Liver Index (FLI) score is one of the best-validated scores for predicting steatosis. The BMI, AST/ALT ratio, and presence of diabetes (BARD) score is reported as having a high negative predictive value, making it especially useful to exclude advanced fibrosis [6,8].

On the other hand, it is well known that obesity is associated with increased morbimortality, namely due to cardiovascular events [9]. Nevertheless, there are different obesity phenotypes that yield different cardiometabolic risks. There is still controversy about defining metabolically healthy and unhealthy obesity [10]. Body fat content is an important marker of metabolic health. Abdominal fat, evaluated by waist circumference and waist-to-hip ratio, is associated with multiple harmful cardiometabolic outcomes [11,12]. Evidence also shows that MS and its associated complications are typically pro-inflammatory states [13]. Namely, in metabolic diseases, there is increased inflammation in the adipose tissue, which leads to the release of pro-inflammatory cytokines and reduction in adiponectin; this causes a disturbance in lipid homeostasis and insulin resistance [14]. The role of inflammation in NAFLD is yet to be understood.

We hypothesized that in patients with morbid obesity, higher waist circumference and waist-to-hip ratio are associated with higher risk of hepatic steatosis and fibrosis. We also hypothesized that, in these patients, higher levels of pro-inflammatory markers are associated with higher risk of hepatic steatosis and fibrosis. Therefore, we aimed to evaluate the association between the risk of NAFLD (through FLI and BARD), and anthropometric and inflammatory parameters (predictors of metabolic status) in patients with morbid obesity.

## 2. Materials and Methods

This study was reviewed and approved by the ethical committee of Centro Hospitalar Universitário de São João, Porto, Portugal (date of approval: April 2021, identification: 80/21). Written informed consent for participation was not required for this study, in accordance with the national legislation and the institutional requirements. Privacy of the patients included was preserved throughout the study.

### 2.1. Study Design and Study Participants

We performed a retrospective observational study evaluating the patients with morbid obesity followed in our tertiary center between January 2010 and July 2020 (total of 3380 patients). Patients missing waist and hip circumferences or FLI and BARD scores (formulas shown below) were excluded, and all other patients were included in the analysis. After applying the former exclusion criteria, 2184 patients, from the 3380 patients evaluated in our institution during the study period, were included in the analysis (Supplementary Table S1 displays the characteristics of included compared to excluded patients).

### 2.2. Clinical and Biochemical Parameters Evaluated

The following parameters were evaluated (collected from the medical records): age, gender, current medication, weight, body mass index (BMI), waist circumference and hip circumference, systolic and diastolic blood pressure. From the biochemical results available, we collected: low-density lipoprotein (LDL) cholesterol, high-density lipoprotein (HDL) cholesterol; and (1) for the score calculation, aspartate transaminase (AST), alanine transaminase (ALT), and triglycerides and (2) as surrogates of inflammation, C-reactive protein, leucocytes count, ferritin. Diabetes was defined by fasting plasma glucose ≥ 126 mg/dL, glycated hemoglobin ≥ 6.5%, 2 h plasma glucose after a 75 g oral glucose tolerance test ≥200 mg/dL [15], or the use of anti-hyperglycemic drugs [15]. Hypertension was defined as systolic blood pressure ≥ 140 mmHg, diastolic blood pressure ≥ 90 mmHg [16], or the use of anti-hypertensive drugs. Dyslipidemia was defined by the use of lipid-lowering agents, serum LDL cholesterol ≥ 160 mg/dL, serum HDL cholesterol < 40 mg/dL, or serum triglycerides ≥ 200 mg/dL [17].

### 2.3. Predictors of Hepatic Fibrosis and Steatosis

FLI and BARD scores were used as predictors of hepatic steatosis and fibrosis, respectively. These were built based on the following formulas:

▪ FLI score: FLI = e^y^/(1 + e^y^) × 100, where y = 0.953 × ln(triglycerides, mg/dL) + 0.139 × BMI, kg/m^2^ + 0.718 × ln(GGT, U/L) + 0.053 × waist circumference, cm–15.745. FLI scores < 30 indicate low risk of hepatic steatosis, 30–60 intermediate risk, and ≥60 high risk [18].
▪ BARD score: BMI ≥ 28 = 1 point; AST/ALT ratio ≥ 0.8 = 2 points, presence of diabetes = 1 point. Low fibrosis risk patients score 0–1 points and higher risk patients score 2–4 points [6].

### 2.4. Outcomes and Statistical Analysis

Continuous variables are described as mean ± standard deviation or median (25–75th percentiles) and categorical variables as proportions (percentages).

We used linear models (unadjusted and adjusted) to evaluate the association of anthropometric measures and inflammatory parameters (considered as continuous variables) with FLI score (considered as a continuous variable). We used ordered logistic regression models to evaluate the association of anthropometric measures and inflammatory parameters with the BARD index (considered as an ordinal variable). The adjusted models included (1) age and sex, and (2) age, sex, diabetes, and dyslipidemia. The choice of covariates was based on prior knowledge of risk factors/confounders and biological plausibility. We performed an exploratory analysis including body mass index (BMI) in the model to evaluate whether the associations were independent from BMI (presented as supplementary). We also performed a stepwise approach model to evaluate the best predictors of FLI, given the associations found in the analysis.

All analyses were conducted with the statistical software package Stata IC version 14.2 (Creator: StataCorp, College Station, TX, USA), using a dataset obtained from the National Institute of Diabetes and Digestive and Kidney Diseases (NIDDK) Data Repository. A two-sided *p*-value < 0.05 was considered statistically significant.

## 3. Results

### 3.1. Baseline Population Characteristics

Clinical and demographic characteristics of the population included in this analysis are presented in Table 1. From the 2184 individuals, around 85% were female. The average age of the population was 43 ± 11 years and BMI was 44 ± 6 kg/m^2^. About 46% of the patients had dyslipidemia, 33% diabetes, and 67% hypertension. Mean waist circumference was 123 ± 25 cm, hip circumference 132 ± 12 cm, and waist-to-hip ratio 0.9 ± 0.2.

### 3.2. Association between FLI and BARD and Anthropometric Parameters

Table 2 summarizes the associations between waist and hip circumferences, and waist-to-hip ratio, with FLI and BARD scores. Our results show a positive association of FLI with waist circumference, hip circumference, and waist-to-hip ratio. These associations were maintained after adjustment in both models. The associations of BARD with the above-mentioned anthropometric measures were statistically significant for waist circumference and waist-to-hip ratio only in the non-adjusted model.

We performed an exploratory analysis adjusting for the parameters included in Model 2, and BMI, and we report a maintained association of FLI with waist circumference and waist-to-hip ratio (Supplementary Table S2).

### 3.3. Association between FLI and BARD and Inflammatory Parameters

**Figure 1:**
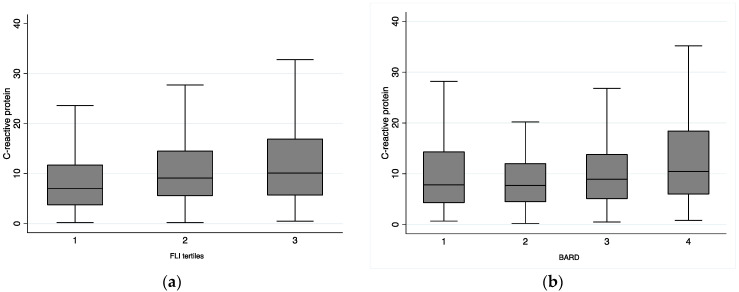
Boxplot displaying C-reactive protein levels (mg/L) according to FLI tertiles (**a**) and BARD (**b**) categories. Abbreviations: FLI, Fatty Liver Index; BARD, BMI, AST/ALT ratio, and presence of diabetes. FLI tertile 1 = 7.41 to 95.56; FLI tertile 2 = 95.56 to 98.68; FLI tertile = 98.68 to 100.

Table 3 displays the associations between FLI and BARD scores and inflammatory parameters (C-reactive protein, leucocytes, and ferritin). Our results show a positive association between both FLI and BARD and C-reactive protein in all the models. There is a positive association between FLI and leucocytes for both adjusted models. Figure 1 represents C-reactive protein levels according to FLI and BARD tertiles.

In our exploratory analysis (Supplementary Table S2), in which we further adjusted for BMI, these associations are maintained.

Considering the associations between FLI and waist circumference, waist-to-hip ratio, C-reactive protein, and leucocytes, we performed a stepwise regression including model 2 and those parameters, to identify the best predictors of FLI. Our final model is presented in Table 4, and it shows that both C-reactive protein and waist circumference remain significant predictors after applying the model.

## 4. Discussion

**Figure 2:**
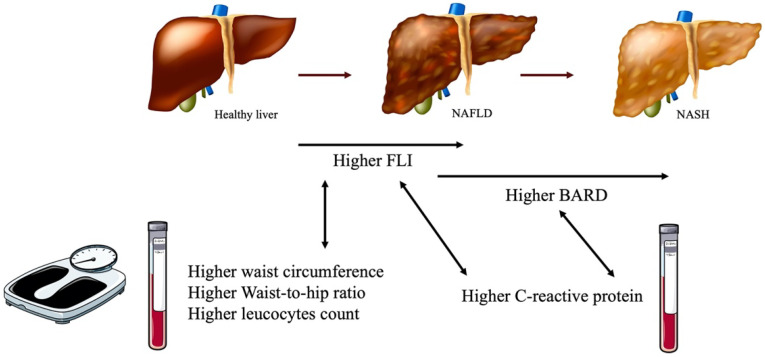
Schematic representation of our main results. Abbreviations: NAFLD, non-alcoholic fatty liver disease; NASH, non-alcoholic steatohepatitis; FLI, Fatty Liver Index; BARD, BMI, AST/ALT ratio, and presence of diabetes. Parts of the figure were adapted with permission from von-Hafe and Borges-Canha et al. [19], and parts were drawn by using pictures from Servier Medical Art (licensed under a Creative Commons Attribution 3.0 Unported License; https://creativecommons.org/licenses/by/3.0/, accessed on 28 June 2022).

This is a retrospective transversal study aiming to evaluate the association between the risk of NAFLD (namely through scores of hepatic steatosis and fibrosis), and both anthropometric parameters, predictors of metabolic status, and pro-inflammatory markers, in patients with morbid obesity (Figure 2 summarizes our main results). Our results point towards a positive association between the risk of hepatic steatosis and waist circumference and waist-to-hip ratio. Additionally, we report a positive association between the risk of both hepatic steatosis and fibrosis and pro-inflammatory markers in patients with morbid obesity. This is a study in a frequently overlooked population and for which data are lacking.

Concerning the anthropometric analysis, our results are in accordance with some previous studies performed in other populations. Sabir et al. also described a positive association between hepatic steatosis and waist circumference and waist-to-hip ratio in patients with obesity [20]. This was also true for lean, overweight, and obese patients studied by Shao et al., and for lean subjects studied by a meta-analysis by Sookoian [21,22]. An increase in the NAFLD risk with an increase in waist-to-height ratio was also described in a large Japanese population [23]. Unlike our results, in which we found no noteworthy correlation between BARD and anthropometric measures, Cholongitas et al. showed that waist circumference had very good discriminative ability between minimal versus significant/severe fibrosis in patients with diabetes and NAFLD [24]. We believe that our results may be limited by the isolated use of this index to assess the risk of fibrosis, and that more studies are needed. Furthermore, we have included in the study the specific population of patients with morbid obesity, for which there is a worrying lack of data.

Regarding the pro-inflammatory profile, our analysis of C-reactive protein is in accordance with Kumar et al., who actually suggested to use high-sensitivity C-reactive protein as a surrogate marker of NAFLD’s severity [25]. Several other studies agree with this line of thought [26,27].

Evidence supports a role of neutrophils (the most populous subset of leucocytes in the circulation) in the pathogenesis of NAFLD, although many issues are yet to be solved [28,29]. In our results, leucocytes are positively and significantly associated with FLI score when the data are adjusted, but no significant association is observed with BARD. On the other hand, ferritin, for which we report no significant associations, was shown to be higher in a population of general American adults with NAFLD [30] and in other populations studied [31,32]. Our lack of significant results on these associations may be biased by the high percentage of missing data on these variables (Supplementary Table S3); however, we cannot exclude that it might be due to the specific characteristics of the population studied.

Overall, our results reinforce the idea that NAFLD, as the hepatic counterpart of MS and linked to other metabolic disorders [2,3], is associated with a metabolically unhealthy obesity, identified in our results by a higher waist-to-hip ratio, a surrogate marker of visceral adiposity, and also a more pro-inflammatory environment. Whether there is a unidirectional or a bidirectional relation between inflammation and NAFLD remains to be established and more studies are necessary to assess this association.

There are limitations inherent to this work that must be acknowledged. Firstly, this is an observational retrospective study and, as such, it is more prone to specific types of bias. For instance, we have missing data concerning the variables studied (Supplementary Table S1), and this may affect our results. Additionally, there are possible confounders to our results that we may have not considered given the design of the study; nevertheless, we trust our models include the most relevant potential confounders. In addition, patients were not submitted to radiological or histological analysis, which could be helpful in the proper diagnosis of NAFLD. Therefore, the results should be interpreted in the context of the risk of NAFLD given by the scores and not definitive NAFLD diagnosis. We believe that the importance of our results, as well as the noteworthy number of individuals included who belong to an often-overlooked population, fairly overcome these limitations.

In conclusion, we demonstrate a positive association between waist circumference and waist-to-hip ratio, and the risk of hepatic steatosis, in patients with morbid obesity, even after adjusting for potential confounders, including BMI. This supports the idea that NAFLD is an indicator of poor metabolic health and is associated with multiple harmful cardiometabolic outcomes. We also show a positive association between pro-inflammatory markers and the risk of both hepatic steatosis and fibrosis. This is in line with the association of NAFLD with a poorer metabolic status and pro-inflammatory profile; the causality of such associations remains to be established, as well as the possible role of inflammation in the progression of NAFLD.

## Figures and Tables

**Table 1:** Clinical and demographic characteristics of the population included (*n* = 2184).

Age, years	42.7 ± 10.5
Feminine sex, *n* (%)	1846 (84.6)
Weight, kg	115.1 ± 18.6
Body mass index, kg/m^2^	43.5 ± 5.8
Waist circumference, cm	123.1 ± 25.1
Hip circumference, cm	131.5 ± 11.8
Waist-to-hip ratio	0.9 ± 0.2
Diabetes, *n* (%)	521 (33.3)
Dyslipidemia, *n* (%)	974 (45.6)
Hypertension, *n* (%)	1136 (66.9)
Smoker *, *n* (%)	307 (15.3)
C-reactive protein, mg/L	8.4 [4.9, 14.0]
Leucocytes, ×109/L	8.1 ± 3.2
Ferritin, ng/mL	87 [39, 166]
AST, U/L	22 [18, 28]
ALT, U/L	24 [17, 35]
GGT, U/L	27 [19, 41]
BARD, *n* (%)	
1	331 (22.1)
2	221 (14.8)
3	666 (44.5)
4	278 (18.6)
FLI	97.4 [93.9, 99.1]

AST: aspartate transaminase, ALT: alanine transaminase, GGT: gamma-glutamyltransferase. * Current or previous smoker. Values are shown as mean ± standard deviation or as median [interquartile range].

**Table 2:** Association of waist and hip circumferences and waist-to-hip ratio with FLI and BARD scores. Model 1—adjusted for age and sex; model 2—adjusted for age, sex, BMI, diabetes, and dyslipidemia. Abbreviations: FLI, Fatty Liver Index; BARD, BMI, AST/ALT ratio, and presence of diabetes.

	FLI		BARD	
	β	*p*-Value	OR	*p*-Value
**Waist circumference, cm**				
Non-adjusted	0.10 (0.09, 0.11)	<0.01	0.99 (0.98, 0.99)	0.016
Model 1	0.31 (0.29, 0.33)	<0.01	1.00 (0.99, 1.01)	0.329
Model 2	0.30 (0.28, 0.32)	<0.01	1.00 (0.99, 1.00)	0.335
**Hip circumference, cm**				
Non-adjusted	0.22 (0.20, 0.25)	<0.01	1.00 (0.99, 1.01)	0.888
Model 1	0.22 (0.20, 0.25)	<0.01	1.00 (0.99, 1.01)	0.943
Model 2	0.22 (0.19, 0.24)	<0.01	1.00 (0.99, 1.01)	0.752
**Waist-to-hip ratio**				
Non-adjusted	8.68 (6.85, 10.52)	<0.01	0.26 (0.09, 0.73)	0.011
Model 1	28.80 (25.06, 32.54)	<0.01	2.25 (0.61, 8.26)	0.220
Model 2	27.48 (23.48, 31.48)	<0.01	0.57 (0.15, 2.24)	0.425

**Table 3:** Association between inflammatory markers and FLI and BARD scores. Model 1—adjusted for age and sex; model 2—adjusted for age, sex, BMI, diabetes, and dyslipidemia. Abbreviations: FLI, Fatty Liver Index; BARD, BMI, AST/ALT ratio, and presence of diabetes.

	FLI		BARD	
	β	*p*-Value	OR	*p*-Value
**C-reactive protein, mg/L**				
Non-adjusted	0.16 (0.11, 0.21)	<0.01	1.02 (1.01, 1.04)	<0.01
Model 1	0.17 (0.12, 0.22)	<0.01	1.03 (1.01, 1.04)	<0.01
Model 2	0.14 (0.09, 0.19)	<0.01	1.02 (1.00, 1.04)	0.031
**Leucocytes, ×10^9^/L**				
Non-adjusted	0.11 (−0.01, 0.22)	0.063	0.97 (0.93, 1.01)	0.109
Model 1	0.19 (0.06, 0.32)	<0.01	0.99 (0.95, 1.04)	0.734
Model 2	0.24 (0.11, 0.38)	<0.01	0.96 (0.01, 1.01)	0.097
**Ferritin, ng/mL**				
Non-adjusted	0.01 (0.01, 0.02)	<0.01	1.00 (1.00, 1.00)	0.07
Model 1	0.01 (−0.00, 0.01)	0.098	1.00 (1.00, 1.00)	0.611
Model 2	0.002 (−0.01, 0.01)	0.631	1.00 (1.00, 1.00)	0.839

**Table 4:** Final regression model after stepwise approach (FLI predictors). Abbreviations: FLI, Fatty Liver Index.

	FLI	
	β	*p*-Value
C-reactive protein, mg/L	0.05 (0.01, 0.10)	0.026
Waist circumference, cm	0.31 (0.28, 0.37)	<0.01
Sex	1.05 (−0.28, 2.39)	0.123
Age, years	0.03 (−0.02, 0.07)	0.236
Diabetes	0.22 (−0.81, 1.24)	0.679
Dyslipidemia	1.94 (1.02, 2.86)	<0.001

## Data Availability

The raw data supporting the conclusions of this article will be made available by the authors, without undue reservation.

## Supplementary Materials

The following supporting information can be downloaded at: https://www.mdpi.com/article/10.3390/biomedicines10102416/s1, Table S1: Clinical and demographic characteristics of included vs excluded patients (n=2184); Table S2: Association of waist and hip circumferences and waist-to-hip ratio with FLI and BARD scores. Exploratory analysis adjusted for age, sex, BMI, diabetes, and dyslipidaemia. Abbreviations: FLI, Fatty Liver Index; BARD, BMI, AST/ALT ratio and presence of diabetes; Table S3: Missing data per variable. Abbreviations: FLI, Fatty Liver Index; BARD, BMI, AST/ALT ratio and presence of diabetes.
