# Supplementary material for: Waist-to-Hip Ratio and Inflammatory Parameters Are Associated with Risk of Non-Alcoholic Fatty Liver Disease in Patients with Morbid Obesity
Source: Biomedicines. 2022 Sep 27;10(10):2416. doi: 10.3390/biomedicines10102416 (PMC9598594; doi:10.3390/biomedicines10102416)
Supplement: Supplementary file 1 [file biomedicines-10-02416-s001.zip › biomedicines-1876490-supplementary.pdf]

Supplementary Table S1. Clinical and demographic characteristics of included vs excluded patients (n = 2184).

	**Included Patients (n = 2184)**	**Excluded Patients (n = 1196)**	***p* Value**
Age, years	42.7 ± 10.5	44.2 ± 10.9	**0.001**
Feminine sex, n (%)	1846 (84.6)	999 (83.5)	0.43
Body Mass Index, kg/m^2	43.5 ± 5.8	43.4 ± 6.1	0.49
Waist circumference, cm	123.1 ± 25.1	120.4 ± 13.5	0.15
Hip circumference, cm	131.6 ± 11.8	130.1 ± 12.7	0.11
Waist-to-Hip ratio	0.9 ± 0.2	0.9 ± 0.1	0.45
Diabetes, n (%)	521 (33.3)	234 (34.9)	0.47
Dyslipidaemia, n (%)	974 (45.6)	437 (43.4)	0.24
Hypertension, n (%)	1136 (66.9)	440 (62.8)	**0.05**

Supplementary Table S2. Association of waist and hip circumferences and waist-to-hip ratio with FLI and BARD scores. Exploratory analysis adjusted for age, sex, BMI, diabetes, and dyslipidaemia.

	**FLI**		**BARD**	
	**β**	***p* Value**	**OR**	***p* Value**
**Waist circumference**, cm	0.24 (0.21, 0.26)	**<0.01**	0.99 (0.98, 1.00)	**0.045**
**Hip circumference**, cm	0.02 (-0.01, 0.06)	0.205	0.99 (0.97, 1.00)	0.206
**Waist-to-Hip ratio**	23.99 (20.57, 27.40)	**<0.01**	0.54 (0.14, 2.12)	0.380
**C-reactive protein**, mg/L	0.05 (0.001, 0.10)	**0.045**	1.02 (1.00, 1.04)	**0.018**
**Leucocytes**, x10^9/L	0.17 (0.06, 0.29)	**<0.01**	0.96 (0.91, 1.01)	0.089
**Ferritin**, ng/mL	0.00 (-0.00, 0.01)	0.256	1.00 (1.00, 1.00)	0.838

Abbreviations: FLI, Fatty Liver Index; BARD, BMI, AST/ALT ratio and presence of diabetes.

Supplementary Table S3. Missing data per variable.

**Variable**	**Number Missing**	**Percent Missing**
Weight	0	0
Waist circumference	3	0.1
Hip circumference	112	5.1
FLI	39	1.8
BARD	688	31.5
C-reactive protein	878	40.20
Leucocytes	1049	48.03
Ferritin	1744	79.9

Abbreviations: FLI, Fatty Liver Index; BARD, BMI, AST/ALT ratio and presence of diabetes.
